# Management-Related Traffic as a Stressor Eliciting Parental Care in a Roadside-Nesting Bird: The European Bee-Eater *Merops apiaster*

**DOI:** 10.1371/journal.pone.0164371

**Published:** 2016-10-05

**Authors:** Julio Blas, Teresa Abaurrea, Marcello D’Amico, Francesca Barcellona, Eloy Revilla, Jacinto Román, Martina Carrete

**Affiliations:** 1 Department of Conservation Biology, Estación Biológica de Doñana (CSIC), Américo Vespucio, 41092, Seville, Spain; 2 Department of Physical, Chemical and Natural Systems, Pablo de Olavide University, Ctra. Utrera km 1, 41013, Seville, Spain; Hungarian Academy of Sciences, HUNGARY

## Abstract

Traffic is often acknowledged as a threat to biodiversity, but its effects have been mostly studied on roads subjected to high traffic intensity. The impact of lower traffic intensity such as those affecting protected areas is generally neglected, but conservation-oriented activities entailing motorized traffic could paradoxically transform suitable habitats into ecological traps. Here we questioned whether roadside-nesting bee-eaters *Merops apiaster* perceived low traffic intensity as a stressor eliciting risk-avoidance behaviors (alarm calls and flock flushes) and reducing parental care. Comparisons were established within Doñana National Park (Spain), between birds exposed to either negligible traffic (ca. 0–10 vehicles per day) or low traffic intensity (ca. 10–90 vehicles per day) associated to management and research activities. The frequencies of alarm calls and flock flushes were greater in areas of higher traffic intensity, which resulted in direct mortality at moderate vehicle speeds (≤ 40 km/h). Parental feeding rates paralleled changes in traffic intensity, but contrary to our predictions. Indeed, feeding rates were highest in traffic-exposed nests, during working days and traffic rush-hours. Traffic-avoidance responses were systematic and likely involved costs (energy expenditure and mortality), but vehicle transit positively influenced the reproductive performance of bee-eaters through an increase of nestling feeding rates. Because the expected outcome of traffic on individual performance can be opposed when responses are monitored during mating (*i*.*e*. negative effect by increase of alarm calls and flock flushes) or nestling-feeding period (*i*.*e*. at least short-term positive effect by increase of nestling feeding rates), caution should be taken before inferring fitness consequences only from isolated behaviors or specific life history stages.

## Introduction

The development of motorized vehicles and the expansion of road networks have contributed to an increase in the anthropogenic impacts currently affecting most habitats worldwide [1, 2]. Roads and traffic often result in wildlife casualties [3, 4], facilitate the introduction of exotic species [5, 6] and reduce the availability of suitable habitats through barrier effects and fragmentation [7, 8] thereby reducing biodiversity.

Management of human disturbance requires a prior understanding of the mechanisms by which anthropogenic stressors promote changes in individual behavior, and how these may affect population distribution and abundance [8, 9]. Birds are often selected as study models to understand the impacts of traffic, whit some species displaying behavioral plasticity [10] and possibly benefitting from traffic [11], whereas other species suffer negative effects on activity patterns and use of space [12], territorial and sexual behavior [13], and also parental care [14]. Several bird species also suffer elevated stress levels due to traffic impact [15], potentially reducing individual fitness and long-term population viability [16].

Despite a growing interest, a vast majority of the studies assessing the effects of traffic stressors on wildlife have been performed on roads exposed to relatively high transit intensity, typically above 1000 vehicles per day. Although protected areas often restrict public access to roads to minimize anthropogenic impacts, management and research activities also involve the transit of vehicles. Because such traffic intensity is relatively low compared to the above referenced studies, their effects on wildlife behavior and individual performance are generally neglected. This provides grounds for a paradox that has been rarely tested: that low levels of conservation-related traffic can actually become a perturbation, potentially jeopardizing the preservation of biodiversity.

With the aim of assessing whether relatively low traffic intensity (less than 100 vehicles per day) can be considered a conservation concern, we quantified the behavioral responses of roadside-nesting European bee-eaters *Merops apiaster* Linnaeus, 1758 to the traffic associated with management and research activities in Doñana National Park (southwestern Spain). The particularities of this bird population offer a powerful model to study the effects of traffic on wildlife behavior for at least two reasons. First, because the flat landscape lacks natural soil cliffs (typically selected by bee-eaters for nesting elsewhere [17, 18]), birds readily dig their nesting burrows in the slight slopes of the ditches of unpaved roads (thus literally breeding beside vehicle-transit areas). Second, because access to most areas within Doñana is restricted to authorized management staff and researchers, who use roads following specific spatio-temporal patterns which entail traffic-exposed areas (with more traffic intensity during rush-hours of workdays) and traffic-free areas (without traffic-intensity variation; [19]). For this reasons we can (*i*) perform an *a priori* selection of sampling locations differing in traffic intensity, and also (*ii*) perform an *a priori*, quasi-experimental sampling design aimed at comparing behaviors within and between days differing in traffic intensity, without performing *ad-hoc* manipulations (see *e*.*g*. [20, 21] for related sampling designs).

Our approach was organized in two main hypotheses. First (*i*), we suggest that traffic can be perceived by individuals as a threat comparable to a predator. This potential threat will elicit behaviors (such as alarm calls and flock flushes) aimed to avoid the potential risk (risk-disturbance hypothesis [16, 22]). Second (*ii*), we hypothesize that bee-eaters would adjust nestling feeding rates to traffic-intensity variation, with fewer visits to the nest according to greater traffic intensity (nestling-feeding hypothesis). This prediction is an offset of the risk-disturbance hypothesis based on ample evidence that wildlife exposed to stressful stimuli require shifting energy and time away from behaviors such as foraging [23, 24] and parental care [14, 25].

## Materials and Methods

### Study area and Study species

The study was carried out between May and July 2012 in Doñana National Park, a Biosphere Reserve and UNESCO World Heritage Site considered one of the most important wetlands worldwide [26]. The main habitats are marshlands, Mediterranean scrublands and coastal sand dunes [27]. The access to the road-network of the National Park is restricted to management staff and authorized researchers. Vehicle speed limit within the National Park is 40 km/h.

The European bee-eater is a medium-sized (50 g) bird that preys on flying insects (mainly Hymenopterans but also Coleopterans and other orders) caught on the wing [17, 28]. Bee-eaters are monogamous and colonial breeders, nesting inside burrows excavated in sloped cliffs [18], although in Doñana they use nearly flat sandy soils [29] and road ditches. This is a migratory species present in Doñana between March and August: building nests in April-May, laying one clutch (4–7 eggs) in May-June, and feeding the nestlings in June-July. The species shows little sexual dimorphism, and both sexes incubate and care for the nestlings [17]. In Doñana, bee-eaters nest in the Mediterranean scrublands, showing colonial aggregations along the ditches of some park roads. For this study we used 7 roadside-colonies composed of 10–20 nests each, all of them in Mediterranean scrublands. The first two colonies were located along unpaved roads with very low traffic intensity (< 2 vehicles per day according to [19]), and therefore they will be further referred to as traffic-free colonies. The five other colonies were located along the gravel road leading to the research station. Since the road crossing these colonies is exposed to the highest intensity of traffic among unpaved park roads (spring average = 80.9 vehicles per day [19]) these colonies will be further referred to as traffic-exposed.

### Data collection

#### Traffic-pattern survey

In order to have a detailed confirmation of the spatio-temporal dynamics of the traffic affecting our study areas, we placed two automatic vehicle counters (TRAFx Vehicle Counter Generation III) beside the roads crossing our traffic-exposed and traffic-free colonies. Automatic counters recorded the number of vehicles passing per 1-hour intervals during the first two weeks of May.

#### (i) Risk-disturbance hypothesis

During the first two weeks of May, when bee-eaters were engaged in nest digging and socio-sexual activity, we also performed 1-hour-long behavioral-observation sessions from camouflage blinds in two traffic-exposed and two traffic-free colonies. Each colony was sampled no more than once a day, between 10:00 and 19:00, on 6–7 different days (total N = 26 observation sessions). We recorded the total number of birds (counted every other minute), and the occurrence of alarm calls, flock flushing events, passing vehicles, and passing predators (raptors and carnivores within 100 meters). A flock flushing event was considered only for the sudden take-off of all the birds present in the colony or at least 10 birds simultaneously, followed by rapid spiral upwards (described in [17] as panic-flight). Socio-sexual activities (*e*.*g*. nest digging, nest defense, aggressions, nuptial feeding, copulations) often involved individual birds, couples or small groups taking off and perching back near their burrows, however, these were not considered flock flushes. Walking transects were performed upon finalization of the observation sessions with the aim of recording road-kills along the road segments adjacent to the colonies.

#### (ii) Nestling-feeding hypothesis

During the first two weeks of July, when bee-eaters were feeding nestlings, we monitored parental feeding rates in traffic-exposed (N = 75) and traffic-free (N = 29) nests from the 7 focal colonies. Feeding events were recorded using a small video camera (GoPro HD Hero^®^) placed on the ground, 50–70 cm from the nest entrance. Although bee-eaters readily resume their normal breeding activities within minutes (typically within 30–60 minutes) following slight modifications of the nest vicinity with new objects (*e*.*g*. artificial perches, marking flags, traps; authors’ personal observations), we placed dummy cameras 24 hours prior to data collection. The resulting 30-minutes-long video-clips were visualized to count the number of feeding events, which always involved one adult carrying a prey upon arrival and leaving the nest without it. All the recordings were performed in the afternoons, allowing us to establish comparisons between traffic rush-hours (14:00–15:00) and post-rush-hours (15:00–20:00) as well as between days differing in traffic intensity (*i*.*e*. workdays *vs*. weekends).

### Data analysis

We used an information-theoretic approach to assess the support provided by our data to several *a priori* defined competing hypotheses (Table 1). Model selection was performed using Akaike Information Criterion corrected for small sample sizes, AICc [30]. Within a given set of models we first calculated ΔAICc_*i*_ as the difference between the AICc of model *i* and that of the best model (*i*.*e*. the model with the lowest AICc). As a rule of thumb, a ΔAICc_*i*_ < 2 suggests substantial evidence for the model; values between 3 and 7 indicate that the model has considerably less support, whereas a ΔAICci > 10 indicates that the model is very unlikely [30]. We also quantified the plausibility of each model as being the best approximation using Akaike weights, *w* [30]. When there was more than one best model (*sensu* ΔAICc_*i*_ < 2) we used model averaging (MuMIn package of R [31]), except when interactions were contained in any of the selected best models, as this would result in biased parameter estimates for variables included in the interaction term. Once the model-averaged estimates and standard errors (SE) were calculated, we used confidence intervals to assess the magnitude of the effect. We considered that a given effect received no, weak or strong support when the 95% confidence interval (CI) strongly overlapped zero, barely overlapped zero, or did not overlap zero, respectively. All statistical analyses were conducted in R 3.1.2 [32].

**Table 1:** Candidate models aimed at explaining: risk-avoidance behaviors (frequency of *alarm calls* per hour = A; frequency of *flock flushes* per hour = B) and factors affecting parental *feeding rate* (C and D).

Models	Fixed terms	k	AICc	ΔAICc	*w*	ER
***Alarm calls*** (events per hour; N = 26)						
A1	*dh*	4	114.8	2.0	0.16	2.7
A2	*dh*, *vehicles*	5	113.0	0.2	0.38	1.1
A3	*dh*, *predators*	5	117.6	4.8	0.04	11.0
A4	*dh*, *vehicles*, *predators*	6	112.8	0.0	0.42	1.0
***Flock flushes*** (events per hour; N = 26)						
B1	*dh*	4	109.7	4.2	0.10	8.2
B2	*dh*, *vehicles*	5	109.8	4.3	0.09	8.6
B3	*dh*, *predators*	5	112.8	7.3	0.02	38.5
B4	*dh*, *vehicles*, *predators*	6	105.5	0.0	0.79	1.0
***Feeding rate*** (events per 30min; N = 104)						
C1	*dh*	5	682.7	5.3	0.066	14.2
C2	*dh*, *area*	6	677.4	0.0	0.934	1.0
***Feeding rate*** (events per 30min; N = 75)						
D1	*dh*	5	522.5	3.2	0.066	4.9
D2	*dh*, *week days*	6	520.8	1.4	0.158	2.0
D3	*dh*, *rush*	6	520.9	1.5	0.151	2.1
D4	*dh*, *week days*, *rush*	7	519.4	0.0	0.323	1.0
D5	*dh*, week days*rush	8	519.5	0.1	0.302	1.1

The last four columns show the parameters allowing selection of the best models within any given set. Smaller AICc values suggest a better fit of the model to data while penalizing for complexity (k, number of estimated parameters). The best supported models (ΔAICC < 2) are highlighted in grey. AICc weights (*w*) indicate the conditional probability of being the best supported model. Evidence Ratio (ER) is the ratio of *w*, comparing the best supported model with every competing one. Legend: *dh* = date and hour; *area* (2-level factor: traffic-exposed *vs*. traffic-free); *week days* (2-level factor: workdays *vs*. weekends); *rush* (2-level factor: rush-hours *vs*. post-rush-hours).

#### Traffic patterns

We confirmed the spatio-temporal patterns of traffic using data from automatic vehicle counters. First, we used all vehicle counts recorded between 07:00h and 22:00h (daylight hours, comprising 93.5% of all the registered traffic) to assess whether *traffic* intensity when bee-eaters are active (*i*.*e*. frequency of vehicles per hour, zero inflated model, logarithmic link function, negative binomial error distribution) differs between traffic-exposed and traffic-free *areas* and along the *week days*. The latter explanatory variable was included as a fixed factor with two levels (workday *vs*. weekend). We considered the independent, additive and interactive effects of these variables.

Then, using information collected by automatic vehicle counters between 14:00 and 20:00 (*i*.*e*. the same time frame when feeding rates were subsequently measured) we tested if traffic also differed across time within days. The dependent variable was again the *traffic* intensity (*i*.*e*. frequency of vehicles per hour, logarithmic link function, Poisson error distribution) while the explanatory variables were *area*, *week days* and *rush-hours*, a fixed factor with two levels: rush-hours (14:00–15:00) and post-rush-hours (15:00–20:00). We also considered the 2-way interactions between rush-hours and the other terms to assess the prediction that rush-time effects are more marked in traffic-exposed areas compared to traffic-free areas, and during workdays compared to weekends. Zero inflated and GLM models were fitted using pscl and Stats packages in R, respectively.

#### (i) Risk-disturbance hypothesis

We previously assessed the prediction that the study areas *a priori* defined as either traffic-exposed or traffic-free actually differed in their exposure to traffic but not in the amount of natural predators. We fitted models where the number of *vehicles* or the number of *predators* recorded per hour during direct observation sessions were considered as dependent variables (logarithmic link function and negative binomial error distribution) and the *area* where each colony was located was included as a fixed factor with two levels (*i*.*e*. traffic-exposed colonies *vs*. traffic-free colonies). *Colony identity* was incorporated as a random term. Models were fitted using the glmmADMB package of R [33].

Then, we assessed whether the frequency of risk-avoidance behaviors depends on the amount of traffic and/or predators by fitting as dependent variables the number of *alarm calls* and the number of *flock flushes* (logarithmic link function and Poisson error distribution) recorded per hour. Competing models included number of *predators* and number of *vehicles* recorded per hour as the main explanatory variables and *date* and *hour* as potentially confounding variables. To control for the non-independence between observations, we included *colony identity* as a random term.

#### (ii) Nestling-feeding hypothesis

The hypothesis that *feeding rate* (feeds per 30 minutes; logarithmic link function, truncated negative binomial error distribution) differs between traffic-exposed and traffic-free areas was assessed by including *area* as an explanatory variable (set C, Table 1). Sampling effort in traffic-exposed areas was larger than in traffic-free areas (N = 75 and N = 29, respectively) because we aimed at assessing week day and rush-time effects on feeding rates in areas where such temporal differences in traffic were predictably most marked. To assess whether the results from the latter models were affected by this unbalanced sampling design, we randomly selected a subsample of 29 observations from traffic-exposed areas. Randomizations were repeated 1000 times, allowing calculation of an average value for model parameters as well as their 95% CIs.

Finally, we assessed the hypothesis that temporal changes in feeding rates depend on the temporal patterns of traffic (set D, Table 1). *Feeding rate* was again the dependent variable (logarithmic link function, truncated negative binomial error distribution), and the explanatory variables were *week days* and *rush-hours*, considering their additive and interactive effects. *Colony identity* was included as a random term, and *date* and *hour* as potentially confounding variables. Models were fitted using the glmmADMB package in R.

### Ethic statement

Espacio Natural de Doñana (Consejería de Medio Ambiente y Ordenación del Territorio; Junta de Andalucía) authorized the fieldwork of the present study (projects CGL2012-32544 MINECO-FEDER Funds and 511/2012 OAPN-MAGRAMA).

## Results

### Traffic patterns

**Fig 1:**
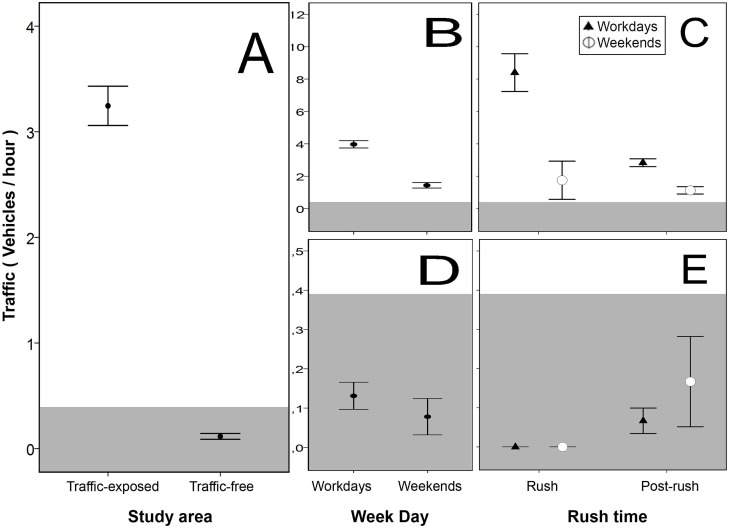
Spatio-temporal variability in daytime traffic intensity (vehicles per hour between 07:00 and 22:00h). (A) Average values in traffic-exposed and traffic-free areas. Traffic intensity across week days and according to rush time are presented separately for traffic-exposed (B-C) and traffic-free (D-E) areas. For ease of interpretation, the grey background indicates traffic intensity below 0.4 vehicles per hour. Bars represent means ±SE.

Data collected from automatic vehicle counters confirmed that traffic-exposed areas suffered thirty times more traffic intensity than traffic-free areas (Fig 1A). On a daily basis, 54.1 vehicles were recorded in traffic-exposed areas (SE = 7.37; range = 9–91), whereas only 2.1 vehicles in traffic-free areas (SE = 0.6; range = 0–8). In the traffic-exposed areas, traffic intensity during workdays was more than twice the amount recorded during weekends (Fig 1B); and traffic intensity was also almost three times higher at rush-hours compared to post-rush-hours during workdays, but not during weekends (Fig 1C). In traffic-free areas, traffic intensity during both workdays and weekends was extremely low (below 0.4 vehicles per hour; Fig 1D and 1E).

### (i) Risk-disturbance hypothesis

The prediction that the study *areas* selected as either traffic-exposed or traffic-free differed in the frequency of *vehicles* but not in the frequency of *predators* was supported by two models with AIC weights of 0.98 and 0.79 respectively, indicating strong to moderate support for being the best models of their sets.

Bee-eaters responded to predators (black kites *Milvus migrans* in all the observations; N = 14 events during 26 hours, all sites combined) with alarm calls in 88% of the occasions, flock flushes in 88% of the occasions or both risk-avoidance behaviors simultaneously in 81% of the occasions. Vehicle passage (N = 42 events during 26 hours of direct observation, all sites combined) elicited the vocalization of alarm calls in 62% of the occasions, flock flushes in 100% of the occasions or both risk-avoidance behaviors simultaneously in 62% of the occasions. Although no road fatalities were recorded in the traffic-free colonies, four birds were found road-killed in the traffic-exposed colonies.

The frequency of *alarm calls* was best explained by two plausible models, containing the frequency of *vehicles* or alternatively the frequency of *vehicles* plus the frequency of *predators* (Table 1A). A consensus model containing the effect size of traffic and predators averaged across these two best models indicated a traffic effect with relatively high support (Tables 1A and 2A). The frequency of *flock flushes* was best explained by a single model containing the frequencies of *vehicles* and *predators* as explanatory terms (Table 1B), suggesting a positive effect of both variables (Table 2B) with relatively high support (*i*.*e*. near 0.80 weight; Table 1B).

**Table 2:** Parameter estimates (±SE) for the terms contained in the best supported models explaining: risk-avoidance behaviors (A and B) and factors affecting parental feeding (C and D).

Dependent variable (Models)	Explanatory Variables	Best supported models (in ascending AICc values)				Model averaged Estimate (±SE)	NCM	RI
		Estimate (±SE)	Estimate (±SE)	Estimate (±SE)	Estimate (±SE)			
*Alarm calls*	Intercept	-	-	-	-	1.42 (0.72)**	-	-
(A4, A2)	*Date*	-	-	-	-	-0.04 (0.02)	3	1
	*Hour*	-	-	-	-	-0.02 (0.04)	3	1
	*Predators*	-	-	-	-	0.20 (0.23)	1	0.43
	*Vehicles*	-	-	-	-	0.19 (0.07)**	2	0.84
*Flock flushes*	Intercept	1.60 (0.66)**	-	-	-	-	-	-
(B4)	*Date*	-0.05 (0.02)	-	-	-	-	-	-
	*Hour*	-0.06 (0.04)	-	-	-	-	-	-
	*Predators*	0.62 (0.18)**	-	-	-	-	-	-
	*Vehicles*	0.27 (0.05)**	-	-	-	-	-	-
*Feeding rate*	Intercept	3.49 (1.19)**	-	-	-	-	-	-
(C2)	*Date*	-0.03 (0.02)	-	-	-	-	-	-
	*Hour*	-0.02 (0.09)	-	-	-	-	-	-
	*Area*	-0.66 (0.24)**	-	-	-	-	-	-
*Feeding rate*	Intercept	-1.20 (2.32)	-0.91 (2.31)	2.55 (1.54)	-1.85 (2.39)	-	-	-
(D4, D5, D2, D3)	*Date*	-0.02 (0.03)	-0.03 (0.03)	-0.03 (0.03)	-0.01 (0.03)	-	4	1
	*Hour*	0.33 (0.18)*	0.32 (0.18)*	0.01 (0.01)	0.36 (0.19)*	-	4	1
	*Week days*	-0.41 (0.22)*	-1.53 (0.81)*	-0.43 (0.22)*	-	-	3	0.84
	*Rush*	-0.65 (0.33)**	-0.76 (0.34)*	-	-0.69 (0.35)*	-	3	0.83
	Week days*Rush	-	0.69 (0.47)	-	-	-	1	0.32

When evidences supported more than one candidate model (*i*.*e*. ΔAICc < 2), model-averaged estimates (±SE) are presented with their associated relative importance (RI) and number of containing models (NCM). When one model included an interaction term, all the competing best models are shown ordered in ascending AICc value. For zero inflated models, estimates for the zero and count models (ZM and CM respectively) are presented. Model identities follow the same notation as in Table 1 (see legend therein for a description of variables).

**95% CI does not overlap zero;

*95% CI slightly overlaps zero.

### (ii) Nestling-feeding hypothesis

**Fig 2:**
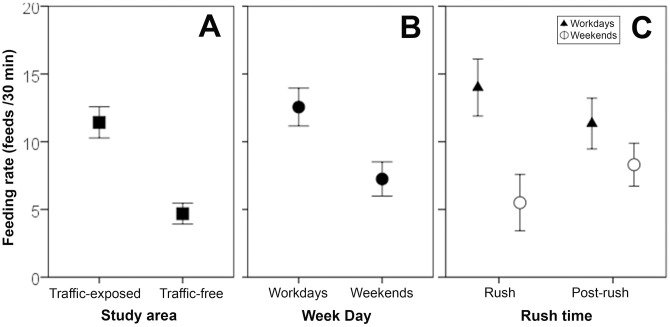
Spatio-temporal variability in feeding rates (feeds/30min). (A) Data from traffic-exposed and traffic-free colonies. Feeding rates across week days (B) and according to rush time (C) correspond to traffic-exposed colonies. Bars represent means ±SE.

*Feeding rates* were higher in traffic-exposed compared to traffic-free areas (Fig 2A), and model C2 received strong support for being the best of its set (*w* > 0.9; Table 1). The randomization procedure performed on 1000 model sets with balanced sample sizes supported this result, with an average AICc value of 357.7 (SE = 0.70) for models containing *area* compared to 360.6 (SE = 0.72) for models excluding *area* (average ΔAICc = 3.1 ± 0.2 SE). Within traffic-exposed areas, the recorded *feeding rates* were higher during workdays compared to weekends (Fig 2B) and higher at rush-hours compared to post-rush-hours, but only during workdays (the opposite pattern occurred during weekends; Fig 2C). Regarding model selection, there were four alternative best models according to AICc, which suggested an effect of *week days* and *rush-hours* on feeding rates (Tables 1D and 2D). Although the 95% CIs for the estimates of these terms slightly overlapped zero (except for *rush-hours*, which showed no overlap) they were consistently negative, supporting higher *feeding rates* during workdays and rush-hours.

## Discussion

In the present study, we confirmed the risk-disturbance hypothesis for a roadside-nesting bird: the European bee-eater. The surveyed individuals systematically perceived relatively low traffic intensity as a threat comparable to a predator, eliciting risk-avoidance behaviors such as alarm calls and flock flushes. On the other hand, our nestling-feeding hypothesis was rejected, because parental feeding rates paralleled changes in traffic intensity, but contrary to our predictions. Indeed, parental feeding rates were highest in traffic-exposed nests, during working days and traffic rush-hours. Several possible mechanisms could explain these surprising findings: prey facilitation by traffic, unmatched cues, energy demand increase, and interference-displacement behaviors.

### (i) Risk-disturbance hypothesis: Traffic as a threat

The positive association between traffic intensity and both alarm calls and flock flushing frequencies suggest that motorized traffic is perceived by European bee-eaters as a threat. Alarm calls are typically used when detecting predators and in response to conspecific distress calls [34]. Alarm calls often lead to flight responses and the retreat of nestlings inside burrows [34], involving energetic costs to signalers and receptors [35]. Moreover, flock flushes consistently occurred in response to approaching vehicles, suggesting absence of habituation to a threat that sometimes resulted in fatal outcomes and likely imposed less immediate costs such as larger energy expenditure.

### (ii) Nestling-feeding hypothesis: Effects on parental care

The results regarding alarm calls and flock flushes provide empirical support for the risk-disturbance hypothesis, just as previously recorded in other bird species but rarely concerning a traffic-related disturbance [16, 22]. However, traffic did not exert a negative effect on parental feeding rates. In fact our results suggested the opposite association, with bee-eaters providing more food to nestlings in traffic-exposed colonies, and showing greater feeding rates during the days of the week experiencing more traffic and during traffic rush-hours. Differences in traffic intensity among study areas were revealed by automatic vehicle counters as well as by our direct observations sessions. Furthermore, our direct observations suggested a similar predator pressure in traffic-exposed and traffic-free colonies, and no other disturbance factors were detected in any of the study areas. Although we cannot rule out the possibility that uncontrolled factors differing among study areas could affect feeding rates, the latter also paralleled temporal changes in traffic intensity within study areas, providing additional support for a positive effect of traffic.

Previous studies have reported greater foraging efficiency in birds along roads or roadsides (*e*.*g*. [11]), but to our knowledge no study has shown a positive effect of traffic intensity *per se*. At least four alternative, non-mutually exclusive mechanisms may explain this association. First (*1*), road transit could elevate the availability or detectability of food resources, improving the foraging efficiency of adults (facilitation hypothesis [11, 36]). A traffic-mediated increase in the abundance or detectability of flying insects was not visually evident to us, and it was not obvious that birds flushed by vehicles went foraging along the road (potentially in the wake of vehicles). However, other bee-eater species have been reported to feed on insects flushed by wild and domesticated mammals, and to forage in association with large birds, humans and motorized vehicles [37, 38, 39]. A second (*2*) possibility is that bee-eaters are attracted to traffic because this stimulus is an effective hunting cue in locations other than our study site (unmatched-cue hypothesis). The lack of asphalt pavements in the study roads generates conspicuous clouds of dust behind passing vehicles, and birds could have learned to use this cue for detecting foraging patches in their African winter areas (*i*.*e*. herds of African ungulates or running cars where flushed insects may abound; see references above). A third (*3*) potential explanation is that greater frequency of risk-avoidance behaviors results in higher energy demands in traffic-exposed nestlings (*e*.*g*. through repeated retreat of nestlings inside burrows or elevated corticosterone levels) triggering a higher feeding frequency by adults (energy-demand hypothesis). Finally (*4*), it is also possible that passing vehicles interrupt the normal progression of adults’ daily activities and disturbed birds redirect their behavior towards nestlings’ feeding (interference-displacement hypothesis). For example, adults hunting for self-maintenance could deliver prey to nestlings because conspecific alarm calls (elicited by car passage) triggered their return to the colony.

### Predicting consequences on health and fitness

Despite our results suggesting that bee-eaters adjust their behavior to the amount of traffic, the net consequences on individual survival, health and performance remain unclear. On the one hand, repeated exposure to traffic can result in road fatalities, and could also bring fitness costs to surviving individuals. Extrapolating data from the vehicle counts recorded during daylight hours, traffic-exposed bee-eaters likely experienced 356 flock flushes and 214 alarm call episodes during one single week (compared to 11 flock flushes and 7 alarm calls in traffic-free colonies). In addition to the direct energy demands associated to the alarm responses themselves [35], disturbance stimuli can indirectly affect individual health and fitness through trade-offs between perceived risk and energy intake or lost opportunity costs [40, 41] or through increased corticosterone levels [15]. On the other hand, a positive effect on parental feeding could bring fitness benefits. Feeding rates were more than two times higher in traffic-exposed nests compared to controls, which might result in overall higher offspring quality (*e*.*g*. improved body condition [42]) and quantity [43]. The consequences of traffic on adults’ health and condition may be different depending on the preponderant mechanism mediating the increase in feeding rates. If the facilitation hypothesis underlies our results, adult bee-eaters would optimize hunting efficiency without necessarily increasing hunting effort [36]. However, if any of the other proposed hypotheses (*i*.*e*. unmatched cue, energy demands, and interference-displacement) underlie our results, the greater feeding effort when prey is neither more abundant nor more detectable could lead to a loss in parental mass, increasing the chances of starvation or the susceptibility to parasites and disease [44]. The expected reproductive benefits would then occur at the expense of compromising adult health and condition in birds already suffering the costs of traffic. Disentangling which of the three hypotheses above explain a traffic-related enhancement of feeding rates will have important consequences for management decisions, as it will influence whether or not the studied roadside environments can be considered ecological traps [45]. Whether the final outcome of road traffic is a positive, negative or neutral effect on bee-eaters’ net fitness is therefore difficult to predict, and should be the focus of future *ad-hoc* research.

### Management implications

Most previous research suggests that a given amount of road traffic can determine a barrier (or a filter) to wildlife movement, and therefore that habitat fragmentation may occur above specific traffic-intensity thresholds ([7], but see [8]). Although we do not know the traffic thresholds affecting bee-eater tolerance, nesting colonies in Doñana National Park do not occur along any public regional road (with average traffic above 1000 vehicles per day [19], all of them crossing potentially suitable habitats for breeding bee-eaters). Such traffic thresholds are well above those recorded in our study area, which included the most frequently used routes by managers and researchers but remained below 100 vehicles per day (range ca. 10–90). Since bee-eater colonies are present in our study area (and they persist over the years), we can suppose that the current traffic thresholds produced by managers and researchers in Doñana National Park are not overstepping the species’ tolerance levels. Nevertheless, bee-eaters modified their behavior in response to such low traffic thresholds, showing lack of habituation to approaching vehicles (as suggested by the 100% rate of flush responses) and experiencing road-kill mortality. Three to seven bee-eaters’ road-kills are yearly recorded along the surveyed traffic-exposed colonies (authors’ personal observation), but the actual impact of road-related mortality should be higher due to the rich scavenger community [27] likely removing most road-killed individuals [46, 47]. Mortality risk seems more evident during the mating and nest building period when flock size is typically larger, as birds are distracted by the intense socio-sexual activities of the colonies and flushing distances decrease [17]. In these circumstances the current speed limit of 40 km/h within park boundaries seems inadequate to avoid collisions. Managers should therefore consider reducing speed limits below 40 km/h, at least beside colony locations and during a few weeks per year. Finally, our results illustrate how the same avian population can show contrasting behavioral responses to the same type of disturbance occurring at different times of its life cycle. Because the expected outcome of traffic on individual performance can be opposed if the responses are only monitored during either mating or nestling-feeding period, we recommend caution before inferring net fitness consequences of traffic from isolated behavioral responses or specific life history stages. Similarly, other avian populations or species inhabiting, for example, different habitats might show contrasting behavioral responses to the same traffic thresholds; and therefore we recommend the actualization of specific studies before suggesting the implementation of mitigation measures.
